# Rare recurrence of a rare ovarian stromal tumor with luteinized cells: a case report

**DOI:** 10.1186/1752-1947-5-350

**Published:** 2011-08-04

**Authors:** Tazi El Mehdi, Ismail Essadi, Hind M'rabti, Hassan Errihani

**Affiliations:** 1 Department of Medical Oncology, National Institute of Oncology, Rabat, Morocco

## Abstract

**Introduction:**

Sex cord-stromal tumors of the ovary are uncommon. They behave unpredictably and often have a late recurrence, making counseling, management, and prediction of prognosis challenging.

**Case presentation:**

A 52-year-old Moroccan woman with an sex cord-stromal tumors underwent a bilateral oophorectomy. The histology was unusual but was likely to be a luteinized thecoma with suspicious features for invasion. Seven years later, after a gastrointestinal bleed, a metastasis within the small bowel mucosa was detected. This represents probable isolated hematogenous or lymphatic spread, which is highly unusual, especially in the absence of concurrent peritoneal disease.

**Conclusions:**

To the best of our knowledge, this is the second reported case of an sex cord-stromal tumors recurring in small bowel mucosa and mimicking a primary colorectal tumor. This highlights the diverse nature and behavior of these tumors.

## Introduction

Sex cord-stromal tumors (SCSTs) of the ovary are uncommon neoplasms that account for 5% to 8% of all ovarian malignancies [1]. Their histopathologic appearance and malignant potential are highly variable, making the optimum treatment of these tumors difficult to establish. The group includes granulosa-stromal tumors, fibroma-thecoma, Sertoli-stromal cell tumors, steroid cell tumors, and SCSTs of mixed or unclassifiable type. The unpredictable behavior and often late recurrence of these tumors make counseling of patients about subsequent management and prognosis a major challenge. We present an unusual case of an SCST that, in keeping with the variable behavior of these tumors, was difficult to classify.

## Case presentation

**Figure 1:**
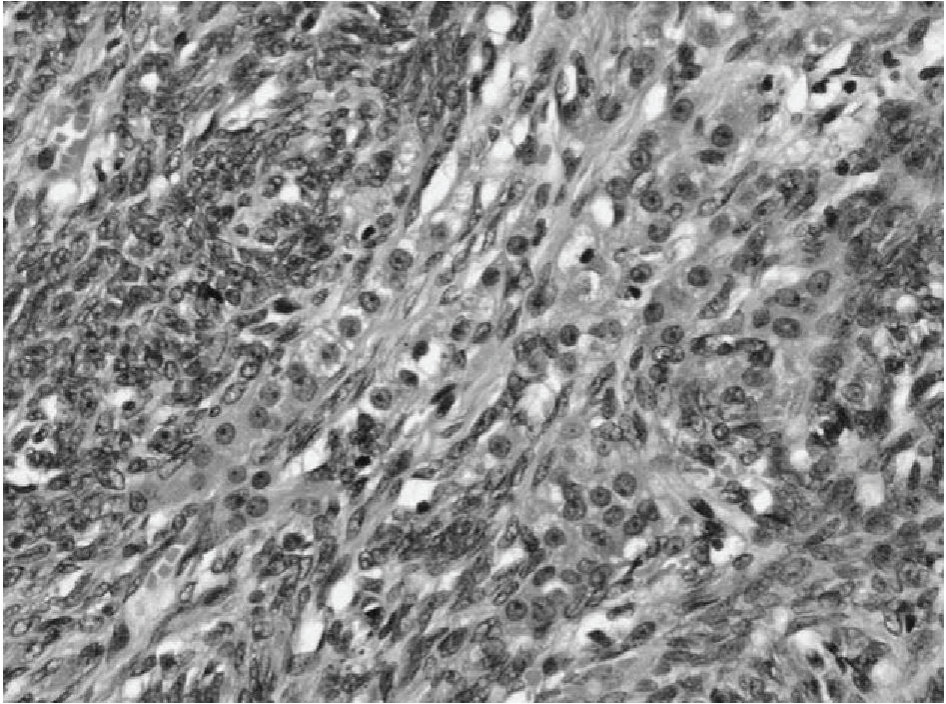
**The histology from the primary ovarian sex cord-stromal type tumor, which was difficult to classify**. In the absence of unequivocal crystals of Reinke, the tumor was classified as a luteinized thecoma. Magnification × 200.

**Figure 2:**
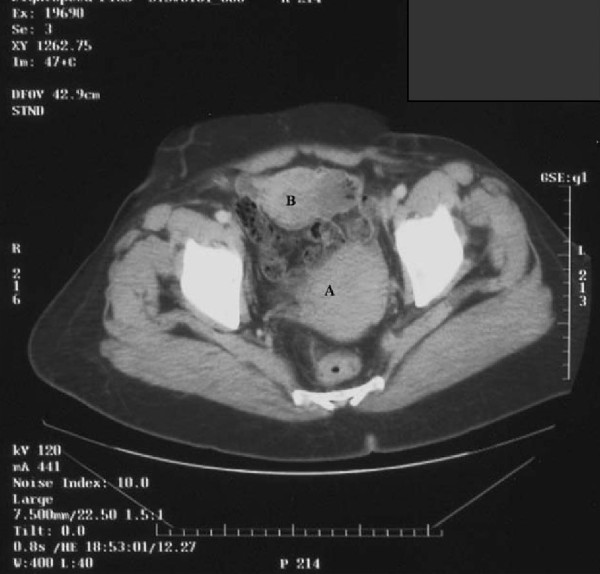
**A computed tomography scan demonstrating a fibroid uterus** (A), anterior to the uterus is an 8x 6x 6 cm mass (B).

**Figure 3:**
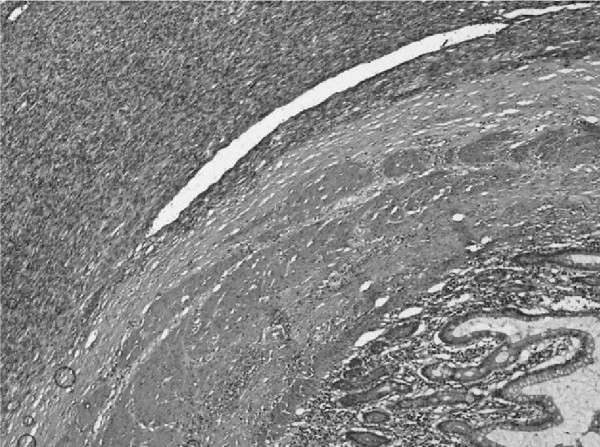
**The histology from the small bowel tumor shows the same histologic features as the primary ovarian lesion**. Normal small bowel villi are also seen. Magnification × 20.

We report the case of a 52-year-old Moroccan woman who had been menopausal for 14 years. She presented with a lower abdominal pain. She had not experienced any postmenopausal bleeding and had no bowel or urinary symptoms. She was a nonsmoker. She had no other significant medical history and no sign of virilization. An examination revealed a smooth cystic mass posterior to the uterus. A transvaginal and transabdominal pelvic ultrasound demonstrated a 9 × 3.7 cm heterogeneous mass in her left adnexa. No vascular flow or ascites was seen, the ovaries did not appear to be separate from the mass. A chest X-ray was normal. Serum CA-125 level was 6 IU/mL. Renal, liver, and hematologic parameters were all in normal range. At laparotomy, a large torted left ovarian cyst with small bowel adhesions to its surface was discovered. Her right ovary appeared normal but was adherent to her small bowel; her uterus was normal. Her liver and omentum appeared normal, and there was no pelvic or abdominal lymphadenopathy. There was a small amount of ascites that was sent for cytology. An oophorectomy was performed with no macroscopic residual disease. An omental biopsy was taken. The histology showed a tumor of sex cord-stromal type in the ovary but the tumor was difficult to classify further (Figure 1). The main left component comprised dense spindle cells interspersed with small groups of cells with a prominent eosinophilic cytoplasm. Crystals of Reinke, a marker of Leydig cell differentiation, were not identified within the majority of these cells, but there was a group at the hilum at the edge of the tumor. In the absence of unequivocal crystals of Reinke, the tumor was reported as a luteinized thecoma. Features such as the mitotic rate were worrying, and the tumor could not be considered benign and we suspected malignancy. The ascites was free of abnormal cells. No vascular invasion was identified. Immunohistochemistry showed that the tumor was positive for inhibin and vimentin. There was no evidence of metastasis in the other specimens received. Our patient made an uncomplicated recovery and was followed up every six months for five years before being discharged. Soon, she presented with a fresh rectal bleeding and melena. She had not experienced any weight loss or change in bowel habit and was otherwise asymptomatic. The initial examination was unremarkable. Upper and lower gastrointestinal endoscopies were performed, and, with the exception of mild gastritis, no abnormality was detected. A computed tomography scan of the abdomen and pelvis confirmed the mass to be within a loop of small bowel with some dilatation of the bowel proximal to this (Figure 2). During laparotomy, a soft intraluminal mass within her ileum was identified. There was no defect in the serosa of the bowel and no evidence of peritoneal or pelvic deposits. A recurrence of the ovarian tumor seemed unlikely. Histology of the lesion showed appearances very similar to those of the previous ovarian tumor except that the endocrine component was more prominent (Figure 3). The gross appearances were very unusual in that there was transmural involvement of the small bowel and an ulcerated polypoid mass protruded into the lumen. The mass was a rare metastatic recurrence of an SCST and appeared to be isolated within the small bowel. Our patient underwent three cycles of a BEP chemotherapy regimen, which consists of bleomycin, etoposide, and cisplatin. She has remained asymptomatic with no clinical evidence of recurrence 27 months after the small bowel resection.

## Discussion

SCSTs vary greatly in prognosis and behavior, depending on the subtype. Therefore, deciding on methods of management for recurrence can be difficult, especially when the tumor is at an unusual site, as in this case. Since disseminated spread is the most common mode of metastasis in ovarian cancer, the gastrointestinal tract is frequently involved. In some advanced cases, determining whether the origin of the tumor is bowel or ovarian can be difficult. The use of immunohistochemical markers such as CK7 and CK20 can assist in the differentiation. The question of tumor origin did not present a problem with this case as the histologic morphology clearly demonstrated a recurrent SCST as opposed to an adenocarcinoma, which could be of ovarian or colonic origin. The majority of gastrointestinal recurrences from ovarian tumors occur via direct transcoelomic spread and invasion of the serosal surface in a centripetal direction. Rose and colleagues [2] demonstrated metastasis relating to small bowel in 43% of autopsies performed for ovarian cancer. However, it is likely that these tumors have mainly serosal involvement and there were no SCSTs in this series [2]. Most tumors involving the mucosa of the bowel will show evidence of associated serosal involvement. Reed and colleagues [3], in a review of 77 autopsy records of ovarian cancer, discovered bowel serosal involvement in 86% and mucosal involvement in 36%. The present case is unusual in that the metastasis appears to have arisen in the mucosa alone, suggesting hematogenous or lymphatic spread. It has been suggested that this spread occurs in epithelial ovarian tumors along bowel wall lymphatic channels, displaying a "buckshot" distribution in which multiple separate foci are seen along a length of bowel mucosa. This lymphatic spread occurs only when there is a high level of intraperitoneal disease, which was not seen in our patient [4]. Some SCSTs are considered to be of low malignant potential, having a low proliferation rate similar to that of borderline tumors of the ovary. Because these SCSTs are slow-growing, they tend to present at an earlier stage compared with epithelial tumors and have an excellent prognosis; overall five-year survival is 79%. Recurrence and metastasis are rare and often late. More than 50% of recurrences occur after more than five years, and 25% occur after 10 years [5]. Others such as thecomas and fibromas generally behave in a benign fashion, although features such as mitotic rate, hemorrhage, and necrosis should be regarded with caution as these recurrences may be better regarded as fibrosarcomas. Both luteinized thecomas and stromal Leydig cell tumors are usually regarded as benign, but in our patient, there were worrying features that were identified on the initial histology. Zhang and colleagues [6] reported a series of 50 ovarian stromal tumors in which luteinized or Leydig cells were present, and four appeared malignant histologically. One patient died early on, one was alive and well at five years, and there was insufficient follow-up on the other two. The authors state that luteinized thecomas and stromal Leydig cells are distinguished only by the presence of crystals of Reinke and that, owing to the difficulty in identifying these structures; some luteinized thecomas may well be unrecognized stromal Leydig cell tumors [6]. Virilization may encourage to search crystals of Reinke. Very few cases of stromal Leydig cell tumors have been reported. In addition to the series of Zhang and colleagues [6], two have been reported in young women [7,8]. Surgical intervention is generally adopted as the primary mode of treatment; this is often conservative in young women wishing to retain their fertility. Age, large tumor size, lymph node involvement, and residual disease are all predictors of poor prognosis [5]. Although surgery is likely to remain the foundation for treatment of SCSTs, chemotherapy has been used as an adjunct in particular cases. Various authors have used chemotherapy in selected cases with advanced stage, significant residual disease, metastasis, or recurrence. Although it is likely that chemotherapy provides improved survival in such cases, the optimum regimen is still unclear. As SCSTs are variable in their histologic appearance, many chemotherapy regimens are assessed by using the most common subgroup, the granulosa cell tumor, and the findings are extrapolated to all SCSTs. In addition, owing to the indolent nature of these tumors, long-term follow-up of any proposed treatment is needed to observe the full effects. Hence, optimizing chemotherapy treatment in these cases is a challenge. A number of regimens - including vincristine, doxorubicin, and cyclophosphamide; BEP; and, most recently, taxanes (paclitaxel or docetaxel) - have been used in the past [9]. A Gynecologic Oncology Group trial used BEP in the treatment of incompletely resected stage II to IV or recurrent SCSTs and found that 69% of primary advanced tumors and 51% of recurrences remained progression-free following treatment, although there were problems of bleomycin-related pulmonary toxicity in a minority of cases [10]. The use of taxanes with or without platinum has been shown to be as effective as BEP with regard to disease-free survival and overall survival. The taxane group experienced less major toxicity (14% mainly hematologic) compared with the BEP group (24% mainly pulmonary fibrosis or neutropenia). It should be noted that, in all of the studies mentioned above, the majority of tumors were adult granulosa cell tumors and so the findings may not be directly applicable to other SCSTs.

## Conclusions

This report further highlights the diverse nature and behavior of this group of tumors. This case demonstrates the difficulty in adopting an evidence-based approach to the treatment and follow-up in these rare cases. The need for careful discussion with the patient about additional treatment, if any, is of paramount importance.

## Abbreviations

BEP: bleomycin, etoposide, and cisplatin; SCST: sex cord-stromal tumor.

## Consent

Written informed consent was obtained from the patient for publication of this case report and any accompanying images. A copy of the written consent is available for review by the Editor-in-Chief of this journal.

## Competing interests

The authors declare that they have no competing interests.

## Authors' contributions

TEM analyzed and interpreted the patient data in regard to its oncological and imaging features and was involved in drafting the manuscript. IE analyzed and interpreted the patient data in regard to its oncological and imaging features. HM was involved in drafting the manuscript. HE gave final approval of the version to be published. All authors read and approved the final manuscript.
